# Everolimus (RAD001) combined with programmed death-1 (PD-1) blockade enhances radiosensitivity of cervical cancer and programmed death-ligand 1 (PD-L1) expression by blocking the phosphoinositide 3-kinase (PI3K)/protein kinase B (AKT)/mammalian target of rapamycin (mTOR)/S6 kinase 1 (S6K1) pathway

**DOI:** 10.1080/21655979.2022.2064205

**Published:** 2022-04-29

**Authors:** Lili Song, Shikai Liu, Sufen Zhao

**Affiliations:** a Department of Obstetrics and Gynecology, The Second Hospital of Hebei Medical University, Shijiazhuang, Hebei, China; b Department of Obstetrics and Gynecology, Cangzhou Central Hospital, Cangzhou, Hebei, China

**Keywords:** Cervical cancer, everolimus, programmed cell death 1 receptor, programmed cell death-ligand 1, PI3K/AKT/mTOR pathway, autophagy, radiosensitivity, T-lymphocytes

## Abstract

Cervical cancer (CC) is the 4^th^ most prevalent malignancy in females. This study explored the mechanism of everolimus (RAD001) combined with programmed death-1 (PD-1) blockade on radiosensitivity by phosphoinositide 3-kinase (PI3K)/protein kinase B (AKT)/mammalian target of rapamycin (mTOR) pathway and autophagy in CC cells. Low-radiosensitive CaSki cells were selected as study objects. After RAD001 treatment, PI3K/AKT/mTOR pathway activation, autophagy, migration and invasion abilities, autophagy-related proteins (LC3-I, LC3-II, and p62), and PD-L1 expression in CC cells were detected. After triple treatment of radiotherapy (RT), RAD001, and PD-1 blockade to the CC mouse models, tumor weight and volume were recorded. Ki67 expression, the number of CD8 + T cells, and the ability to produce IFN-γ and TNF-α in tumor tissues were determined. RAD001 promoted autophagy by repressing PI3K/AKT/mTOR pathway, augmented RT-induced apoptosis, and weakened migration and invasion, thereby increasing CC cell radiosensitivity. RAD001 elevated RT-induced PD-L1 level. RT combined with RAD001 and PD-1 blockade intensified the inhibitory effect of RT on tumor growth, reduced the amount of Ki67-positive cells, enhanced radiosensitivity of CC mice, and increased the quantity and killing ability of CD8 + T cells. Briefly, RAD001 combined with PD-1 blockade increases radiosensitivity of CC by impeding the PI3K/AKT/mTOR pathway and potentiating cell autophagy.

## Graphical abstract

**Figure:**
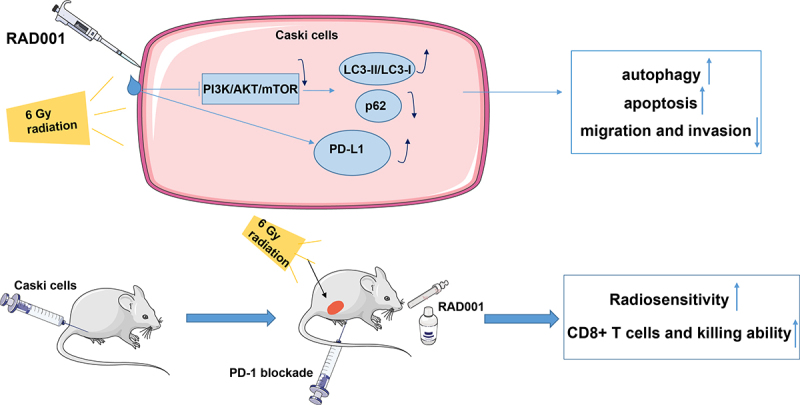

## Highlights

- RAD001 promotes RT-induced CC cell autophagy by blocking PI3K/AKT/mTOR pathway.
- RAD001 inhibits malignant behaviors and increases radiosensitivity of CC cells.
- RAD001 augments RT-induced PD-L1 expression in CC cells.
- RAD001+PD-1 blockade increases radiosensitivity of CC mice.
- RAD001+PD-1 blockade enhances the therapeutic effects of RT.

## Introduction

Cervical cancer (CC) is initiated from the cervix, characterized by vaginal discharge, aberrant vaginal bleeding, and pelvic pain [1]. CC is predominantly classified into 2 prime histopathological subtypes: adenocarcinoma and squamous cell carcinoma (the latter occupies over 75–80% of CC) [2]. It is noteworthy that CC ranks the 4^th^ most prevailing cancer in women and the 7^th^ most prevailing of all human cancers, which contributes to approximately 569,000 new cases and 311,000 deaths in 2018 [3,4]. There are more than 90% of deaths in low and middle-income countries [5]. CC is attributed to diverse risk factors, such as drinking, smoking, unprotected sex, multiple sexual partners, extended use of oral contraceptives, a family history of CC, socioeconomic status, and virus infections [6]. Moreover, nearly all CC cases are caused by human papillomavirus [7]. The primary treatments for CC include surgery (such as radical hysterectomy and pelvic lymphadenectomy), chemotherapy, and radiotherapy [8–10]. Due to nearly half of CC cases diagnosed at a locally advanced stage, surgical treatment is not an effective option for them and radiotherapy is potentially favorable to treat these CC patients [11]. However, the curative effect is hampered by insensitivity to radiotherapy [12]. Herein, we probed into the mechanism to improve the radiosensitivity of CC and expected to provide a reference for the clinical treatment.

Mammalian target of rapamycin (mTOR) is a human serine/threonine kinase, which belongs to the phosphoinositide 3-kinase (PI3K)-associated kinase family that regulates gene expression, cell signaling, metabolism, and autophagy [13]. There is evidence to suggest that the mTOR pathway is activated in CC [14]. Additionally, ionizing radiation (IR) can trigger the activation of the PI3K/protein kinase B (AKT)/mTOR pathway [15]. Monotherapy and the combined treatment with mTOR inhibitors are widely used in clinical and pre-clinical trials in different cancers [16,17]. Natural and synthetic mTOR inhibitors can enhance radiotherapy effects and diminish the radioresistance of CC cells [18]. Everolimus (RAD001) is an important mTOR inhibitor [19,20]. Recent research has documented that RAD001, in combination with other drugs, exerts a synergistic role in preventing gastric cancer by controlling the PI3K/AKT/mTOR pathway [21]. Therefore, RAD001 was selected as the study target to find out valuable strategies for treating CC.

As targeted immune checkpoint inhibitors, programmed death-1/ligand 1 (PD-1/L1) emerge as the key points of neoplasm treatment owing to their good curative effects [22]. The amplification of genes that are related to PD-L1 expression in CC individuals has been unveiled [23]. The PD-1/L1 pathway exerts an oncogenic function, assisting tumors in escaping from immunosurveillance by inversely mediating the proliferation and role of tumor-directed T cells [24]. Anti-PD-1 therapy may strengthen the efficacy of radiotherapy through immunoactivation in the tumor microenvironment [25]. A recent study has reported that Pembrolizumab combined with microRNA-20b-5p enhances tumor cell radiosensitivity by impeding the PD-L1/PD-1 axis, thereby suppressing tumor cell growth *in vivo* [26]. Furthermore, repression of autophagy in tumor cells elevates cell radiosensitivity [27]. The compelling evidence has confirmed the importance of blocking the PD-1 pathway in cancer therapy and the effect of autophagy on radiotherapy in cells. Importantly, nivolumab (PD-1 inhibitor) has a longer overall survival than RAD001 and PD-L1 inhibitors can increase the antitumor effect of RAD001 in renal cell carcinoma [28,29]. However, there are limited reports on the function of RAD001 combined with PD-1 inhibitors in CC treatment. This study decided to use RAD001 in combination with PD-1 blockade to treat tumor cells to observe the effect of drug combination on CC treatment.

Autophagy is an important physiological process of catabolism for cell survival by which cells remove damaged organelles and recover nutrients to maintain homeostasis, which is complexly implicated with cancer since autophagy can lead to the death or survival of cancer cells depending on the different stages of neoplasm development [30,31]. There is evidence to suggest that the induction of autophagy is implicated in the inhibition of CC invasion [32]. Intriguingly, PD-L1 regulates glucose and lipid metabolism, autophagy, and stemness [33]. The PI3K/AKT/mTOR pathway promotes neoplasm proliferation and survival by repressing autophagy [34]. However, whether RAD001 combined with PD-1 blockade mediates radiosensitivity in CC cells by affecting autophagy has not been reported.

In this study, we postulated that RAD001 combined with PD-1 blockade exerted vital functions in CC treatment, to determine whether inhibition of mTOR and immune checkpoints by RAD001 and PD-1 blockade could enhance the radiosensitivity in CC cells, and we preliminarily discussed the mechanism of the combination therapy to provide a new theoretical basis for radiotherapy of CC.

## Materials and methods

### Ethics statement

The animal experiments were conducted under institutional guidelines and protocols involving animals. This study was approved by the ethics committee of the Second Hospital of Hebei Medical University (Approval No. 2021-P005). Considerable efforts were made to minimize the number of animals and their pains.

### Cell culture

Human CC cell lines Hela and CaSki were provided by ATCC (Manassas, VA, USA). Hela cells were derived from cervical adenocarcinoma, and CaSki cells were derived from CC, both with the characterization of epithelial cells-like and adherent growth. Cells were cultured in Dulbecco’s modified Eagle medium and RPMI-1640 containing 10% fetal bovine serum (FBS) (HyClone Laboratories, Logan, UT, USA) at 37°C in an incubator with 5% CO_2_ [12].

### Ionizing radiation (IR)

After pretreatment with or without RAD001, CC cells were exposed to IR by an X-ray linear accelerator (RS2000, Rad Source, Suwanee, GA, USA) at a dose rate of 1.24 Gy/min [35].

### Colony formation assay

CC cells pretreated with or without RAD001 (Calbiochem, LaJolla, CA, USA) were seeded in 6-well plates at 1 × 10^3^ cells/well and were irradiated using different doses of X-ray (0, 2, 4, 6, and 8 Gy), followed by further 2–3 weeks of culture post irradiation. After 20 min of fixation with 4% paraformaldehyde, cells were stained with 0.5% crystal violet for 10 min at room temperature. The colonies containing 50 or more cells per dish were calculated. Finally, the data were fitted to a linear-quadratic model [36] to draw survival curves, thereby assessing the radiosensitivity of these cells. ImageJv1.8.0 software was used for counting.

### Cell grouping and treatment

Relatively radioresistant CaSki cells were selected for *in vitro* study. The CaSki cells were divided into the following 3 groups: blank group; RT group (cells received radiation at a dose of 6 Gy), and RT + RAD001 group (cells received radiation at a dose of 6 Gy after 24 h of treatment with 10 nM RAD001) [28].

### Acidic vesicular organelle (autophagosome) detection

At 72 h post above-mentioned cell grouping and treatment, the cells (5 × 10^5^ cells/mL) were gathered in fluorescence-activated cell sorting (FACS) tubes followed by staining with 5 µg/mL acridine orange (Sigma-Aldrich, Darmstadt, Germany) at room temperature for 15 min. Subsequently, the cells were rinsed with Ca^2+^/Mg^2+^-free phosphate buffer saline (PBS) twice and then immediately analyzed via a FACSCalibur flow cytometer (BD Biosciences, San Jose, CA, USA) [37].

### Cell apoptosis

At 72 h post-irradiation, the cells were adjusted to 5 × 10^5^ cells/mL and then stained using Annexin V-fluorescein isothiocyanate (FITC)/propidium iodide apoptosis detection kits (#PF032, Merck, Kenilworth, NJ, USA). The FACSCalibur flow cytometer (BD Biosciences) was employed to analyze the cell cycle and apoptosis. The results were exhibited as the dot plot [38].

### Reverse transcription quantitative polymerase chain reaction (RT-qPCR)

At 72 h post-irradiation, total RNA was extracted from CaSki cells using the TRIzol reagent (ComWin Biotech, Beijing, China). Additionally, the quality and quantity of RNA at 260 and 280 nm wavelengths were detected using the NanoDrop-2000 ultramicrospectrophotometer (Thermo Fisher Scientific, Waltham, MA, USA). Subsequently, RNA (1 mg) was reversely transcribed into cDNA by a SuperRT cDNA Synthesis kit (Beijing ComWin). qPCR was implemented using SYBR Green qPCR SuperMix (Thermo Fisher Scientific) under a CFX96^TM^ Real-Time PCR Detection System (Bio-Rad Laboratories, Hercules, CA, USA). The conditions of thermal cycling were as follows: pre-denaturation at 95°C for 5 min, then 40 cycles of 95°C for 30s and 60°C for 30s, and final extension at 72°C for 5 min [39]. The 2^−ΔΔCt^ method [40] was performed to normalize the relative expression of genes to glyceraldehyde-3-phosphate dehydrogenase (GAPDH). The primer sequences are presented in Table 1.

**Table 1.** Primer sequences

Name of primer	Sequences
GAPDH-F	5’-TGACTTCAACAGCGACACCCA-3’
GAPDH-R	5’-CACCCTGTTGCTGTAGCCAAA-3’
PD-L1-F	5’-TTTGCTGAACGCCCCATA-3’
PD-L1-R	5’-TGCTTGTCCAGATGACTTCG-3’

GAPDH, glyceraldehyde-3-phosphate dehydrogenase; F, forward; R, reverse; PD-L1, programmed death-ligand 1.

### Flow cytometry

The surface PD-L1 quantitation on CaSki cells was carried out using flow cytometry. The intracellular staining was also used to analyze the PD-L1 expression in cells. Concisely, the collected single-cell suspensions (5 × 10^5^ cells/mL) were stained with peroxidase (PE)-conjugated mouse immunoglobulin G (IgG) isotype control antibody (eBiosceience, San Diego, CA, USA) or anti-mouse PD-L1 antibody. After fixation using Fixation/Permeabilization solution at 4°C for 12 min, cells were rinsed with Perm/Wash buffer (BD Biosciences) followed by staining with PE-conjugated mouse IgG isotype control antibody or anti-mouse PD-L1 antibody at 4°C for 15 min [41]. Anti-mouse PD-L1 antibody (clone 10 F.9G2) was provided by Bio-Xcell (West Lebanon, NH, USA).

### Western blot (WB)

At 72 h post-irradiation, CC tumor tissue homogenate or cells were treated with the cell lysis buffer (KeyGEN, Nanjing, China) and phosphatase inhibitor (Sigma-Aldrich) on ice to prepare cell extracts, boiled for 10 min under reduction conditions, and frozen at −20°C for later usage. Subsequently, 30 μg proteins were separated in 12% sodium dodecyl sulfate-polyacrylamide gel electrophoresis and blotted onto immobilon polyvinylidene fluoride membranes (EMD Millipore, Billerica, MA, USA). After blocking with 5% skim milk in 0.05% Tris-buffered saline with Tween (TBST) for 1 h at room temperature, the membranes were probed with the mouse monoclonal antibodies listed below: anti-PI3K (1:1000, ab86714, Abcam, Cambridge, MA, USA), anti-phosphorylated (p)-AKT (1:1000, ab105731, Abcam), rabbit monoclonal anti-p-mTOR (1:1000, ab109268, Abcam), anti-p-S6 kinase 1 (S6K1; 1:1000, ab59208, Abcam), LC3B (1:1000, ab192890, Abcam), and p62 (1:1000, ab109012, Abcam) overnight at 4°C. Membranes were incubated with rabbit anti-mouse IgG (1:10000, ab6728; Abcam) or goat anti-rabbit IgG (1:10000, ab205718, Abcam) horseradish peroxidase-labeled secondary antibodies for 1 h at room temperature. After washing with TBST, an enhanced chemiluminescence solution (Thermo Fisher Scientific) was adopted to examine the blot [39]. GAPDH acted as the internal control.

### Transwell assay

The Transwell plates (EMD Millipore) were used to assess CC cell invasion and migration abilities at 72 h post-irradiation. CC cells were seeded in Matrigel-coated (for invasion analysis) or uncoated (for migration analysis) chambers at a diameter of 8 mm (BD Biosciences). They were seeded in the apical chamber (2 × 10^4^ cells/well) in the serum-free medium. The basolateral chamber was added with 10% FBS. Non-migrating cells in the upper surface of the filter were discarded after 24 h of incubation. Afterward, 5 fields were arbitrarily chosen to determine the CC cells migrating to the basolateral chamber using the optical inverted microscope.

### In vivo study

Total 40 C57BL/6 female mice (3–4 weeks old, 16.85 ± 2.19 g) were purchased from Tengxin Biotechnology (Chongqing, China). The mice were strictly placed in an air-cleaned laminar flow rack at a constant temperature (24°C ± 2°C) and humidity (50% ± 10%) in a specific pathogen-free environment. The mouse cage, air filter cover, bedding, food, and drinking water were disinfected and then replaced in a sterile environment. RAD001 used here was a solution and it was prepared with 30% propylene glycol and 5% Tween80 (carrier). The solvent of anti-mouse PD-1 was InVivo Pure pH6.5 dilution buffer (BP0101, Bio-Xcell).

All cancer cells were implanted under intraperitoneal injection of 1% pentobarbital sodium (50 mg/kg), and considerable efforts were made to minimize the pain. To establish the xenograft model, CC cells were suspended in Hanks’ balanced salt solution (HBSS), and the right thigh of mice was subcutaneously inoculated with 0.2 mL HBSS (containing 1 × 10^7^ cells). The mice were randomly allocated into 5 groups (N = 8), with the day of the first injection of tumor cells as day 0: (A) control: mice were subjected to mock oral administration or injection on days 11, 13, and 15, under oral administration of the same dose of 30% propylene glycol and 5% Tween80 (carrier), and injection of an equal dose of InVivo Pure pH6.5 dilution buffer; (B) RT: tumors were irradiated with 6 Gy on days 12, 14, and 16; (C) RT + RAD001: mice were orally administrated with 0.25 mg/kg RAD001 on days 11, 13, and 15, followed by 6 Gy irradiation 24 h later [42]; (D) RT + PD-1 blockade: mice were intraperitoneally injected with 200 μg/mouse Anti-PD-1 on days 11, 13, and 15, followed by 6 Gy irradiation 24h later; (E) RT + RAD001 + PD-1 blockade: mice were subjected to simultaneous combined treatment on days 11, 13, and 15 by oral administration of 0.25 mg/kg RAD001 and intraperitoneal injection of 200 μg Anti-PD-1, followed by 6 Gy irradiation 24h later. The tumor size was measured every 3 days based on the formula: Volume = Length × Width^2^/2. Mice were anesthetized by intraperitoneally injecting 1% pentobarbital sodium (50 mg/kg) on day 21, followed by euthanasia and collection of tumor tissues for flow analyses and immunohistochemical detection.

### Radiotherapy (RT)

The mice were placed prone on a body membrane fixation plate in the awake state and irradiated with a linear accelerator 12 MeV electron beam at 600 cGy/min every other day, with an irradiation field of 3 cm in diameter plus 1 cm compensation glue, and a source skin distance of 100 cm. Dose total = 6 Gy × 3 f.

### Detection of the number and killing ability of tumor-infiltrating CD8 + T cells

Acquisition of tumor-infiltrating lymphocytes cells was conducted based on the Percoll gradient [43] as follows: the tumor tissues were weighed, cut into small pieces of about 1–2 mm^3^, and placed in the 50 mL centrifuge tube. The tissues were incubated with collagenase (StemCell Technologies, Vancouver, BC, Canada) for 2 h at 37°C, followed by centrifugation at 200 g for 5 min with the supernatant discarded. Next, 3 mL 70% Percoll (GE Healthcare, Chicago, IL, USA), 40% Percoll, and tumor resuspension solutions were successively added into the 15 mL centrifuge tube. After centrifugation at 200 g for 40 min, the supernatant was removed. The intermediate layer between 70% Percoll and 40% Percoll solution was tumor-infiltrating T cells, which were removed, washed twice with PBS, and added into a flow tube for detection.

The number of CD8 + T cells (CD8+/CD45+) in tumor-infiltrating lymphocytes was measured on the machine using CD3-PE-Cy7/CD8a-PerCP/CD45-PE-Cy7/CD4-FITC four-color reagent. Subsequently, the selected CD8 + T cells were respectively added with interferon (IFN)-γ-PE or tumor necrosis factor (TNF)-α-APC antibodies to determine the number of IFN-γ+ CD8+ cells and TNF-α+ CD8+ cells. Flow-associated fluorescent antibodies CD4-FITC, CD8a-PerCP, CD3-PE-Cy7, CD45-PE-Cy7, IFN-γ-PE, and TNF-α-APC were purchased from Biolegend (San Diego, CA, USA). This study adopted the FACSCalibur flow cytometer (BD Biosciences) and the FlowJo v10 software (FlowJo, Ashland, OR, USA) was used for data analysis.

### Immunohistochemical study

Formalin-fixed paraffin-embedded (FFPE) sections were used for immunohistochemistry. FFPE CC tumor sections were cut (3 μm), deparaffinized in xylene, and rehydrated in a series of gradient alcohols and distilled water. Endogenous peroxidase was blocked by distilled water containing 3% hydrogen peroxide for 5 min. The nonspecific binding was blocked with normal horse serum for 30 min at 37°C [28]. Later, they were diluted with Ki67 (ab16667, Abcam) at 1:500. VECTASTAIN ABC kit (Vector Laboratories, Burlingame, CA, USA) was adopted for detection. Ki67 was quantitated based on the ratio of Ki67-positive cells/total cells.

### Statistical analysis

All data were processed using SPSS 21.0 statistical software (IBM Corp. Armonk, NY, USA). Measurement data were exhibited as mean ± standard deviation (SD). The data were all presented in normal distribution via examination using the Shapiro-Wilk (W test). An independent sample *t*-test was used for data analysis between two groups. One-way analysis of variance (ANOVA) was employed for comparisons among multiple groups. Tukey’s multiple comparisons test was implemented for the post hoc analysis. A linear-quadratic model was adopted to draw survival curves of radiation dose under the formula: Y = exp (- (a*x + b*(x^2)). The confidence interval value was 95%, and the *P* < 0.05 indicated statistically significant.

## Results

The present study aimed to investigate the molecular mechanism of RAD001 combined with PD-1 blockade to enhance the radiosensitivity of CC by impeding the PI3K/AKT/mTOR pathway. CC cells Caski were subjected to RT treatment and it was found that RT inhibited CC cell autophagy by activating the PI3K/AKT/mTOR pathway. RAD001 promoted CC cell autophagy by impeding PI3K/AKT/mTOR pathway activation, enhanced RT-induced apoptosis, and reduced CC cell migration and invasion, thereby improving CC cell radiosensitivity. Moreover, RAD001 augmented RT-induced PD-L1 expression in CC cells. Additionally, in the CC mouse models, the combination of RAD001 and PD-1 blockade potentiated the inhibitory effect of RT on tumor growth, decreased the number of Ki67-positive cells, increased the radiosensitivity of CC mice, and enhanced the number and killing ability of CD8 + T cells.

### RAD001 promoted autophagy by blocking the RT-induced PI3K/AKT/mTOR pathway in CC cells

**Figure 1.**
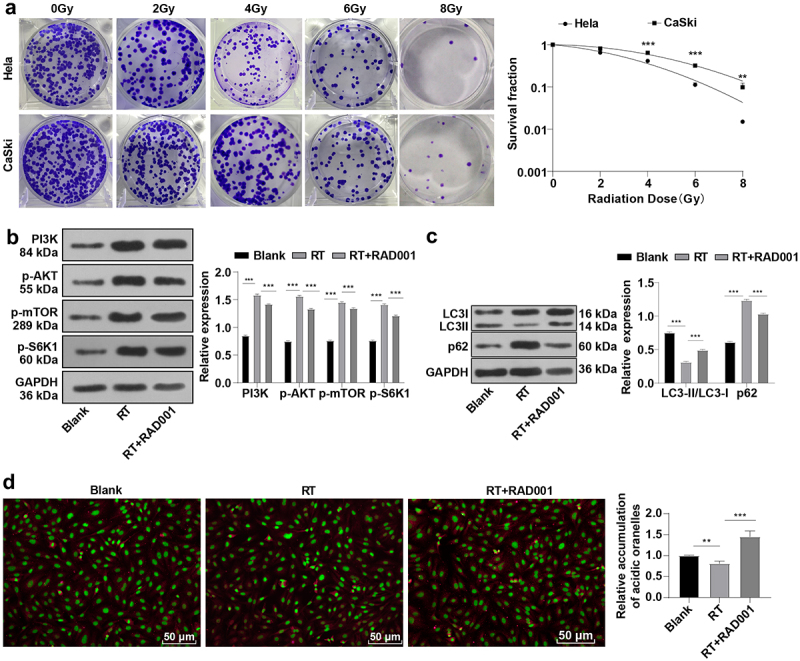
RAD001 promoted autophagy by blocking the RT-induced PI3K/AKT/mTOR pathway in CC cells. (a) The radiosensitivity of CaSki and Hela cells was assessed using colony formation assay; (b) The expression levels of PI3K/AKT/mTOR pathway-related proteins were detected by WB; (c) Autophagy-related proteins LC3-II, LC3-I, and p62 expression levels were measured by WB; (d) Acidic autophagic vesicles in CC cells were detected by the fluorescence microscope and flow cytometry, indicating the formation of acidic vesicular organelle 24 hours after RT, and the values were normalized to that in the blank group. The radiation dose was 6 Gy, and the cell experiment was conducted 3 times. Data were expressed as mean ± SD, the independent sample *t*-test was used for comparisons between the two groups, and one-way ANOVA was used for comparisons among multiple groups. Tukey’s multiple comparisons test was adopted for the post hoc analysis. ***P < 0.001, **P < 0.01.

The colony formation assay showed that CaSki cells treated with different radiation doses had higher cell survival ability than Hela cells (Figure 1(a)), indicating higher radiosensitivity of Hela cells than that of CaSki cells (*P* < 0.05). Therefore, we selected CaSki cells with lower radiosensitivity as study subjects for subsequent experimentation. It is noteworthy that IR activates the PI3K/AKT/mTOR pathway, and blocking this pathway can increase radiosensitivity by enhancing autophagy [44–46]. WB was used to detect the expression levels of the PI3K/AKT/mTOR pathway-related proteins and autophagy-related proteins in CaSki cells prior to and post the radiation. The results revealed elevated levels of PI3K, p-AKT, and p-mTOR in CC cells after 6 Gy radiation compared with the unirradiated group (*P* < 0.001, Figure 1(b)), illustrating that RT induced the activation of the PI3K/AKT/mTOR pathway in CC cells. As a protein kinase, mTOR can phosphorylate key components of the protein synthesis mechanism, such as S6K1 [47]. Hence, the phosphorylation level of S6K1 was measured, which was increased with the activation of mTOR (*P* < 0.001, Figure 1(b)). Importantly, detecting the conversion of LC3-I to LC3-II is the most common method to determine the cell autophagy activity 48, and the quantity of LC3-II is closely related to the number of autophagosomes and is a key indicator of cell autophagy activity. On the contrary, the level of p62 protein is inversely proportional to autophagy activity and is an accessory protein for detecting autophagy activity [49]. WB assay revealed that CC cells after 6 Gy radiation had decreased LC3-II/LC3-I levels and increased p62 expression compared with the unirradiated group (all *P* < 0.01, Figure 1(c)). In addition, acidic vesicular organelle detection suggested that radiation markedly reduced the amount of acidic autophagic vesicles in CC cells (*P* < 0.01, Figure 1(d)). To investigate whether RAD001 could impede the RT-activated PI3K/AKT/mTOR pathway, 10 nM RAD001 was added into CC cells 24 h prior to RT. The experimental results demonstrated that RAD001 remarkably decreased the expression levels of PI3K, p-AKT, p-mTOR, and p-S6K1 in CC cells relative to that in the RT group (all *P* < 0.001, Figure 1(b)), manifesting the inhibitory effect of RAD001 on the PI3K/AKT/mTOR pathway and S6K1 phosphorylation in CC cells. Simultaneous detection of autophagy activity in CC cells illustrated that compared with the RT group, RAD001 treatment prominently elevated LC3-II/LC3-I ratio, lowered p62 protein expression (*P* < 0.001, Figure 1(c)), increased the number of acidic autophagic vesicles in CC cells (*P* < 0.01, Figure 1(d)). The above results elicited that RT could repress autophagy in CC cells by activating the PI3K/AKT/mTOR pathway, whereas RAD001 boosted autophagy by inhibiting the RT-stimulated PI3K/AKT/mTOR pathway in CC cells.

### RAD001 promoted the RT-induced CC cell apoptosis and inhibited migration and invasion

**Figure 2.**
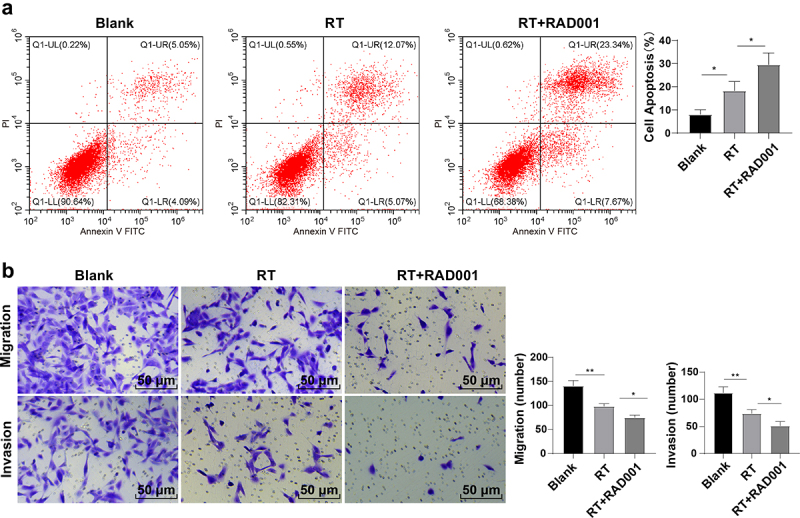
RAD001 promoted the RT-induced CC cell apoptosis, inhibited migration and invasion. (a) CC cell apoptosis rate was detected by flow cytometry; (b) CC cell migration and invasion abilities were assessed using Transwell assays. The radiation dose was 6 Gy, and the cell experiment was conducted 3 times. Data were presented as mean ± SD. One-way ANOVA was used for data comparisons between multiple groups and Tukey’s multiple comparisons test was adopted for the post hoc analysis.**P < 0.01, *P < 0.05.

Flow cytometry was performed to further analyze the apoptosis level (Q1-UR region represented late apoptotic cells, Q1-UL represented necrotic cells, Q1-LL region represented living cells, and Q1-LR represented early apoptotic cells). Compared with the unirradiated group, the apoptosis rate of CC cells after radiation was considerably elevated and RAD001 further amplified the apoptosis rate (all *P* < 0.01, Figure 2(a)), indicating the promotion effect of RAD001 on RT-induced CC cell apoptosis. Transwell assays revealed that RT or RAD001 combined with RT both significantly reduced the migration and invasion of CC cells, and the combined treatment had augmented this decrease (all *P* < 0.01, Figure 2(b)). To sum up, RAD001 could promote RT-induced CC cell apoptosis and suppress migration and invasion.

### RAD001 increased radiosensitivity of CC cells

**Figure 3.**
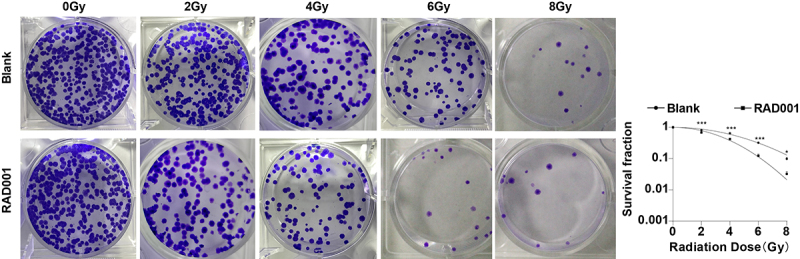
RAD001 increased radiosensitivity of CC cells. Colony formation assay was employed to determine the radiosensitivity of CC cells. The cell experiment was conducted 3 times. Data were exhibited as mean ± SD and independent sample *t*-test was implemented for data comparisons between the two groups. ***P < 0.001, *P < 0.05.

Previously we had confirmed that RAD001 could promote RT-induced apoptosis and inhibit migration and invasion in CC cells, so we further evaluated the effect of RAD001 on radiosensitivity of CC cells by colony formation assay. The results unveiled that, in comparison with untreated CC cells, the RAD001-treated CC cells had a diminished survival fraction after different doses of radiation (*P* < 0.01, Figure 3), demonstrating that RAD001 treatment markedly enhanced the radiosensitivity of CC cells.

### RAD001 augmented RT-induced PD-L1 expression in CC cells

**Figure 4.**
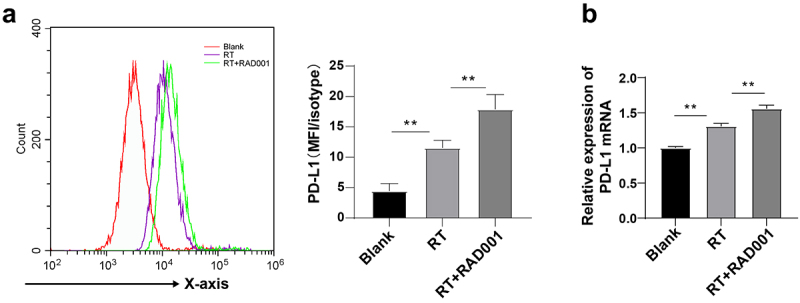
RAD001 augmented RT-induced PD-L1 expression in CC cells. (a) PD-L1 expression in CC cells was detected using flow cytometry; (b) PD-L1 level in CC cells was measured by RT-qPCR. The radiation dose was 6 Gy, and the cell experiment was conducted 3 times. Data were displayed as mean ± SD. One-way ANOVA was employed for data comparisons between multiple groups and Tukey’s multiple comparisons test was used for the post hoc analysis. **P < 0.01.

PD-1 and its ligand PD-L1 are important immune checkpoint proteins and autophagy is one of the internal functions that affect PD-L1 [50]. Compared with PD-L1-downregulated cells, PD-L1-upregulated cells are more sensitive to autophagy inhibitors. To explore whether RT and inhibition of AKT/PI3K/mTOR pathway affected PD-L1 expression in CC cells, PD-L1 expression in CC cells was detected using flow cytometry and RT-qPCR. The results unraveled that PD-L1 was remarkably upregulated in RT-induced CC cells relative to untreated CC cells, and RAD001 prominently enhanced the upregulation of PD-L1 in CC cells (*P* < 0.01, Figure 4(a)), which was consistent with the RT-qPCR results (Figure 4(b)). Briefly, mTOR inhibitor RAD001 could increase the RT-induced PD-L1 level in CC cells.

### RAD001 combined with PD-1 blockade increased radiosensitivity of CC mice

**Figure 5.**
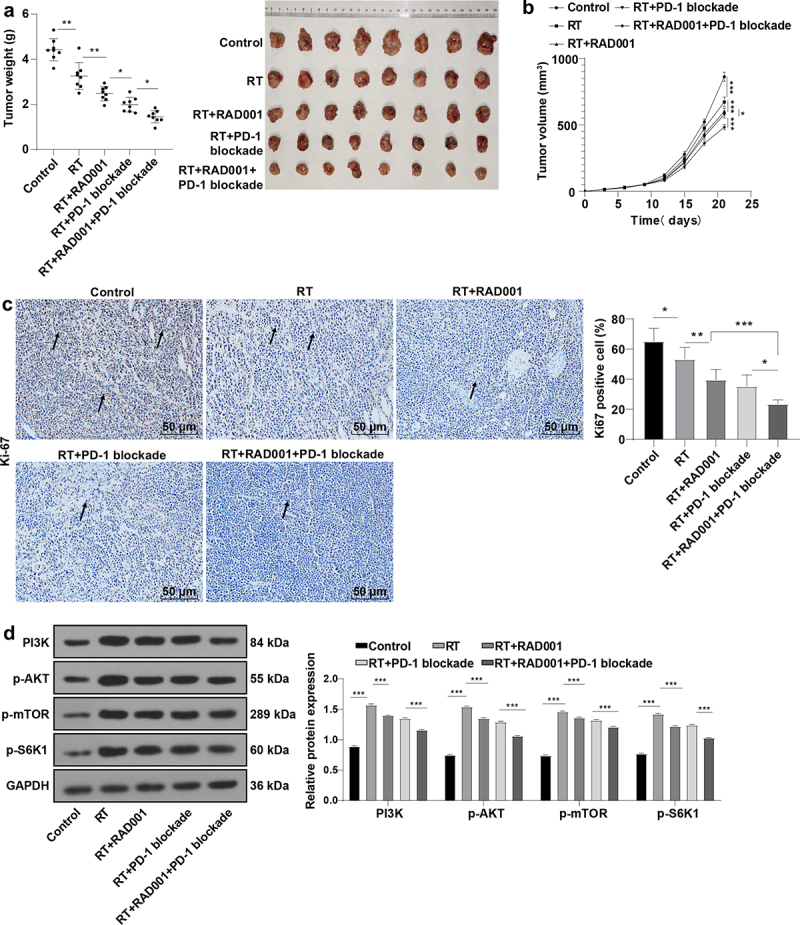
RAD001 combined with PD-1 blockade increased radiosensitivity of CC mice. (a-b) Tumor weight and volume of CC mice were recorded; (c) The expression of Ki67-positive cells was determined by immunohistochemical staining; (d) mTOR signaling pathway-related proteins were detected by WB. The radiation dose was 6 Gy, N = 8. Data were expressed as mean ± SD. One-way ANOVA was performed for data comparisons between multiple groups and Tukey’s multiple comparisons test was employed for the post hoc analysis. ***P < 0.001, **P < 0.01, *P < 0.05.

Interestingly, PD-1/PD-L1 inhibitors can enhance the anti-cancer effect of RAD001 in renal cancer cells [28,29], and the drug targeting the PD-1/PD-L1 axis combined with autophagy inhibitor provides a new opportunity for tumor treatment. Autophagy inhibitor RAD001 previously had been found to significantly increase CC cell radiosensitivity, and PD-L1 was upregulated after treatment of RAD001 combined with RT. To further examine whether RAD001 combined with PD-1 blockade could elevate CC radiosensitivity, the CaSki xenograft tumor model was established. By detecting the tumor weight and volume of mice, it was found that RT markedly repressed the tumor growth in CC mice compared with the control group, and the inhibitory effect of RT on tumor growth was intensified after RT combined with RAD001 or PD-1 blockade alone, or combined with RAD001 and PD-1 blockade together, with the most inhibitory effect on tumor growth in the triple treatment (all *P* < 0.05, Figure 5(a,b)). Moreover, immunohistochemistry showed that RT combined with RAD001 and PD-1 blockade group had the lowest rate of Ki67-positive cells (all *P* < 0.05, Figure 5(c)). In addition, we detected the mTOR pathway-related proteins by WB analysis (Figure 5(d)), and the results unveiled that RT activated the mTOR signaling pathway, and RT combined with RAD001 or PD-1 blockade impeded the mTOR signaling pathway. Moreover, the triple treatment with RT, RAD001, and PD-1 blockade exerted more predominant inhibitory effects on the mTOR pathway in mice. Collectively, RAD001 combined with PD-1 blockade remarkably enhanced radiosensitivity in CC mice.

### RAD001 combined with PD-1 blockade enhanced the RT-induced CD8 + T cells and killing ability in CC mice

**Figure 6.**
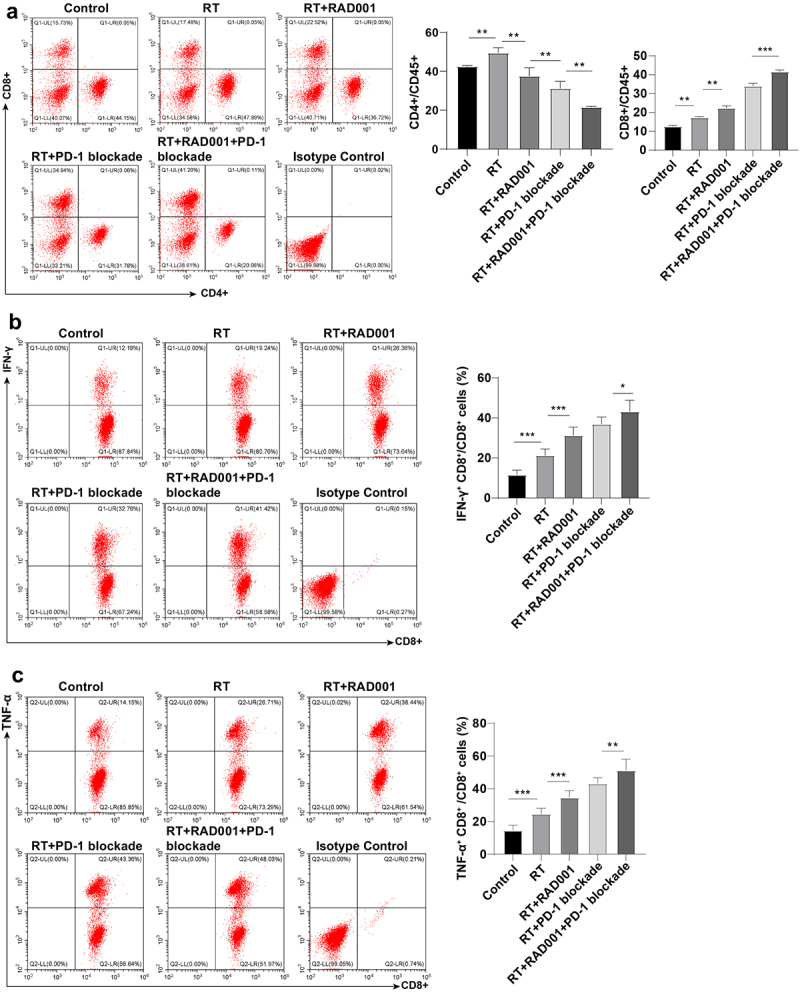
RAD001 combined with PD-1 blockade enhanced RT-induced CD8 + T cell number and killing ability in CC mice. (a) The number of CD8 + T and CD4 + T cells was measured by flow cytometry; (b) The ability of CD8 + T cells to produce IFN-γ was assessed using flow cytometry; (c) The ability of CD8 + T cells to produce TNF-α was evaluated via flow cytometry. The radiation dose was 6 Gy, N = 8. Data were presented as mean ± SD. One-way ANOVA was adopted for data comparisons among multiple groups and Tukey’s multiple comparisons test was implemented for the post hoc analysis. **P < 0.01, *P < 0.05.

Subsequently, the amount of CD8 + T cells in tumor tissues was measured by flow cytometry to investigate the effect of agents combined with RT on lymphocytes. The results revealed that relative to the control group, the number of CD8 + T cells was prominently elevated in CC mice treated with RT alone or RT combined with agents, with the highest number in RT combined with RAD001 and PD-1 blockade group (*P* < 0.05, Figure 6(a)). The analysis of the ability of CD8+ lymphocytes to produce IFN-γ and TNF-α could reflect the CD8+ cell activation and killing ability to tumors. It was statistically observed that the percentages of IFN-γ+ CD8+ and TNF-α+ CD8+ cells were maintained at a low level in the single RT and control group. Relative to the RT+RAD001 group, the IFN-γ+ CD8+ and TNF-α+ CD8+ cells were notably increased in the RT+PD-1 blockade group, and the activation and killing ability of CD8+ cells were greatly augmented in the triple treatment (all *P* < 0.01, Figure 6(b,c)). Collectively, RAD001 combined with PD-1 blockade increased RT-induced CD8 + T cell number and killing ability in CC mice.

## Discussion

CC represents a prevalent malignancy in females worldwide, covering 7.5% of all cancer-associated mortality [51,52]. RT is an accepted treatment for CC, while reoccurrence of CC has emerged after RT owing to low radiosensitivity [53,54]. Additionally, mTOR inhibitor RAD001 possesses an anticancer effect on CC [55]. PD-L1 expression has been documented in most cervical squamous cell cancers, indicating the potential effectiveness of anti-PD-1 therapies for CC [56]. This study evaluated the effect of RAD001 combined with PD-1 blockade on the radiosensitivity of CC

Firstly, CaSki cells were selected as the study subjects due to their low radiosensitivity. The PI3K/AKT/mTOR pathway is tightly implicated in tumorigenesis, cancer cell proliferation, and RT-resistance [39,57]. S6K1 functions as an imperative signaling mediator of cell growth and proliferation in the mTOR signaling pathway [58]. Intrinsically, autophagy is known to play a crucial role in cancer radioresistance [37]. p62 and LC3-II/LC3-I protein levels are important in the process of autophagy [59,60]. After radiation, WB detection showed increased PI3K, p-AKT, p-mTOR, p-S6K1, and p62 and decreased LC3-II/LC3-I levels and acidic autophagic vesicles in CC cells. The activation of the PI3K/AKT/mTOR pathway is linked with cytostatic drug resistance and RT [61], which is also involved in incomplete metabolic responses in CC, but inhibitors of PI3K/AKT may improve the response to chemoradiation [62]. Blocking p62 expression induces the activation of autophagy [63] and reduced LC3-II/LC3-I ratio is synchronized with the decrease of autophagy flux formation [64]. It is noteworthy that radon radiation could increase p-PI3K, p-AKT, and p-mTOR levels, and the anti-tumor effect is accompanied by the inhibition of the PI3K/AKT/mTOR pathway [65–67]. Our results elicited that RT suppressed autophagy by activating the PI3K/AKT/mTOR pathway in CC.

RAD001 engages in the regulation of the PI3K/AKT/mTOR pathway [68–70]. Next, we investigated the relationship between RAD001 and RT-activated PI3K/AKT/mTOR pathway by adding RAD001 after RT. The results revealed that treatment of RAD001 diminished the expression levels of PI3K, p-AKT, p-mTOR, p-S6K1, and p62, and elevated the LC3-II/LC3-I ratio and amount of acidic autophagic vesicles in CC cells. Consistently, RAD001 impedes the expression levels of PI3K, p-AKT, AKT, mTOR, and p-mTOR in epilepsy rats [71]. Mounting evidence has unveiled the promotion effect of numerous anti-tumor drugs on apoptosis and autophagy through the suppression of AKT/mTOR signaling [72]. Altogether, RAD001 triggered autophagy by inhibiting the RT-induced PI3K/AKT/mTOR pathway. The inhibitor of the PI3K/AKT/mTOR pathway combined with radiation raises the radiosensitivity of cancer cells by activating autophagy [73]. Next, we analyzed the role of RAD001 in RT-induced other cellular functions. It was observed that RAD001 treatment significantly augmented the inhibiting effect of RT on migration and invasion and promoted the effect on apoptosis. Subsequent colony formation assay illustrated that the CC cell survival rate was decreased after adding RAD001. In line with our results, RAD001 markedly suppresses cell growth and lowers migration and invasion potentials in oral squamous cell carcinoma [74]. RAD001 is involved in the antitumor process by inhibiting colony formation, proliferation, and migration [75]. Moreover, RAD001 can induce apoptosis by repressing mTOR and exhibits a preventive function against CC [76]. mTOR inhibitors can reduce the tumor resistance to IR and increase the radiosensitivity of different cancer cells [77,78]. Radiation combined with RAD001 may be potent to overcome tumor radioresistance by targeting vascular microvascular endothelial cells in radioresistant tumors [79]. RAD001 + RT + cisplatin could bring about complete response in locally advanced CC [80]. Collectively, RAD001 strengthened the radiosensitivity of CC cells.

The PD-L1/PD-1 axis is vital in the immune escape of CC through suppression of host immune response [81], and PD-L1 expression is related to tumor-infiltrating lymphocytes [82]. Therefore, we explored whether RT and AKT/PI3K/mTOR pathway inhibitors could affect the expression of PD-L1. The results suggested that PD-L1 was upregulated after RT, and a supplement of RAD001 amplified this increase. Anti-cancer treatments including radiation therapy can elevate PD-L1 level in tumor cells [83]. Furthermore, RAD001 is correlated with the increase of PD-L1 expression [84]. Our results elicited that RAD001 could promote the RT-induced PD-L1 level in CC cells.

PD-L1 is a major immune checkpoint protein that impedes immune function by binding to the PD-1 receptor, and immunotherapy targeting PD-1 emerges as standard pharmacological therapy [85,86]. The upregulation of PD-L1 in CC supports the utilization of PD-1/PD-L1 antagonist therapy in patients, and the elevated PD-L1 is linked with poor survival [87]. Then, we assessed the combined effect of RAD001 and PD-1 blockade on the treatment for CC in mice. Single RT or combined with RAD001, PD-1 blockade, or triple treatment prevented the tumor growth and lowered the rate of Ki67-positive cells, with the best effect in triple treatment. In addition, RT combined with RAD001 or PD-1 blockade or the triple treatment inhibited the RT-activated mTOR pathway. Ki67 indicator independently predicts cancer development and is upregulated in malignant cells [88]. PI3K/AKT/mTOR pathway inhibitors combined with RT is a new method to enhance radiosensitivity and for treatment in some different cancers such as CC, and mTOR inhibitors possess a possible function to promote the efficacy of RT [18,89,90]. Anti-PD-1 therapy enhances tumor response to RT [25]. In addition, nivolumab (PD-1 inhibitor) demonstrates a better overall survival than RAD001 in patients with renal cell carcinoma [91–94], and anti-PD-1 pembrolizumab is applied for treating recurrent or metastatic CC [95]. Briefly, RAD001 combined with PD-1 blockade increased radiosensitivity of CC mice.

CD8 T cell mediates cytotoxicity and CD8 cytotoxic T lymphocyte is an important immune cell for targeting cancer [96,97]. Post-RT persistent lymphopenia is considered a poor prognostic factor for CC individuals receiving RT [98]. Preceding research has evidenced that a single high dose of RT can increase the accumulation of CD8 + T cells in tumors [99]. The expression of tumor-infiltrating CD8 + T cells in tumor tissues may reflect the effect of antitumor immunity [100,101]. Meanwhile, CD8 + T cell immune responses are influenced by immunosuppressive factors in the local tumor microenvironment, such as cytokines released by innate immune cells, Treg cells, and myeloid-derived suppressor cells, and signaling/PD-L1 released by immune checkpoint molecules including PD-1 [102,103]. Malignant tumors can evade host immune response via diverse mechanisms, among which although multiple mechanisms contribute to immune system homeostasis, PD-L1 still exerts a pivotal role in the tumor microenvironment [104]. In the present study, our experimental results showed that RAD001 increased RT-induced PD-L1 expression and mediated CD8 + T cell immune responses, manifested by high infiltration of CD8 + T cells, which provided a plausible explanation for the enhanced antitumor effects of RAD001 combined with PD-1 blockade. The activation of TNF-α can increase intracellular killing [105]. CD8 T cells can produce IFN-γ and IFN-γ cells generate cytotoxic molecules [106]. Additionally, RT combined with PD-1 blockade elevated the IFN-γ+ CD8+ and TNF-α+ CD8+ cells, and the triple therapy greatly augmented the activation and killing ability of CD8+ cells. The increased CD8 T cells after RT indicate a good radiation response in patients [107]. mTOR inhibitor can stimulate the cytotoxic CD8 + T-cell with a suitable dose/duration [108]. PD-1 inhibitory is related to the enhanced number of memory CD8 + T cells [109]. *In vitro*, PD-1 blockade potentiates activation of CD4 tumor-infiltrating lymphocytes, as manifested by elevated CD154 level and cytokine generation, contributing to the improvement of dendritic cell maturation and ultimately enhanced proliferation of tumor-specific CD8 T cells [110]. mTOR signaling mediates the fate decision between effector and memory CD8 + T cells [111], and RAD001 increases the caspase-dependent killing ability [112]. Radiation can trigger a prominent supra-additive cytotoxic effect in CC cells when combined with mTOR inhibitors [18]. Conjointly, RAD001 combined with PD-1 blockade elevated the RT-stimulated CD8 + T cell number and killing ability in CC mice.

In conclusion, this study elucidated a novel mechanism of RT+RAD001+ PD-1 blockade on enhancing radiosensitivity in CC by inhibiting the PI3K/AKT/mTOR pathway and promoting autophagy. However, we only simply revealed the effect of RAD001 combined with PD-1 inhibitor on autophagy and radiosensitivity. Autophagy also possesses immunomodulatory functions. In tumor cells, the application of antibodies including anti-PD-1/PD-L1 can trigger autophagy, thus allowing adjacent cells to recycle nutrients and signals and release cytokines and extracellular vesicles. Moreover, autophagy can promote tumor cell survival and reduce chemotherapy effectiveness in a variety of ways. Future studies shall explore the regimen of using immune checkpoint inhibitors in combination with autophagy inhibitors for anti-cancer therapy to improve the therapeutic effectiveness.

## Conclusion

The current study elucidated that RAD001 combined with PD-1 blockade enhanced CC cell autophagy and apoptosis and suppressed migration and invasion by impeding the RT-activated PI3K/AKT/mTOR pathway, which was valuable for improving the radiosensitivity in CC cells.

## Data Availability

All the data generated or analyzed during this study are included in this published article.
